# How *TP53* (almost) became an oncogene

**DOI:** 10.1093/jmcb/mjz061

**Published:** 2019-08-13

**Authors:** Robert A Weinberg

**Affiliations:** Whitehead Institute for Biomedical Research, Ludwig/MIT Center for Molecular Oncology, MIT Department of Biology, Cambridge, MA 02142, USA

Like the histories of many other genes, the discovery and characterization of p53 are associated with some false leads, dead ends, and major detours. The chronicle of p53’s early years is no exception. As is now well known, the protein was actually discovered independently by three groups—those of Arnie Levine, David Lane, and Lloyd Old.

The year 1979 was truly the *annus mirabilis* of p53 research. In March of that year, the laboratory of Lionel Crawford and his then-post-doctoral fellow, David Lane, described the association of p53 with the SV40 large T antigen, against which they had developed an effective immunoprecipitation (Lane and Crawford, 1979). Two months later, two others research groups—those of Arnie Levine whose career we celebrate here and of Lloyd Old—reported the existence of this protein and tumor antigen independently (DeLeo et al., 1979; Linzer and Levine, 1979). This, on its own, was an unusual convergence of three fully independent research efforts occurring essentially simultaneously.

The research in the Lloyd Old Laboratory at Memorial Sloan-Kettering was led by Albert DeLeo. He found that the tumors created by methyl-cholanthrene-induced mouse sarcoma cells elicited an antibody response reactive with a protein of ~ 53 kDa; the same anti-serum detected this protein in a variety of other transformed murine cell lines. He noted that the protein was generally not very immunogenic, that the source of its immunogenicity when expressed by the methyl-cholanthrene-induced sarcoma cells was unclear, and that it was likely a protein of cellular origin, since it was expressed in a variety of murine cancer cell lines tested, some of which had no indication of viral infection (DeLeo et al., 1979).

The subsequent development by the Old Laboratory of an anti-p53 monoclonal antibody led this group to demonstrate its widespread expression in a variety of tumor cell lines, its nuclear localization, and the fact that its elevated expression was associated with the active proliferation of cells. This association of p53 expression with cell proliferation was not observed in certain neoplastic cells, which instead expressed it constitutively. Among other hypotheses, this group speculated that p53 might be involved in initiating DNA synthesis. In another work, they reported in May of 1981 the fact that this protein formed complexes with SV40 large T antigen and the fact that cross-reactive proteins could be found in the tumor cells of other species. Over the next four decades, DeLeo’s group, then in Pittsburgh, pursued p53 as an antigen that might serve as an attractive target for anti-tumor immunotherapy (DeLeo, 1998; Dippold et al., 1981).

By 1980, the protein had been found in a variety of transformed cells including those transformed by Abelson murine leukemia virus. Research on this protein, termed variously p53, p50, even p48, soon exploded, and by 1983 a workshop was held in the UK focusing specifically on p53 and attended by almost two dozen researchers whose laboratories had already made the study of this protein a significant focus of their research agenda. The allure of this protein was irresistible. Its expression in a wide range of cancer cell types held the promise of finally yielding a universally expressed tumor antigen that could serve as a target of anti-tumor immunotherapy.

Already by 1979, the major fork in the road of p53 research was apparent. DeLeo’s group, as said, focused on the role of this protein as a tumor antigen; the other groups—those of Levine and Crawford—turned their attention to the functional role of the p53 protein, specifically its cell-physiologic role in cell transformation. Arnie Levine’s group, soon including Moshe Oren, and that of Lionel Crawford and importantly his trainee David Lane, soon led the charge in the functional characterization of p53 (Lane and Crawford, 1979; Linzer and Levine, 1979).

At this point, it is instructive to recall the mindset of cancer biologists at the time. In 1976, the research in the group of Harold Varmus and Mike Bishop had revealed the existence of the Rous sarcoma virus *src* oncogene and its derivation from the corresponding normal proto-oncogene, *c-src* (Stehelin et al., 1976)*.* It is difficult to overestimate the conceptual impact of this finding, specifically the existence of genes in the normal cellular genome that, when reshaped in various ways, could function as dominant alleles in driving cell transformation. Amidst the subsequent flurry of retrovirus-associated oncogene research, my own group reported in mid-1979 the existence of a dominantly acting oncogene residing in the genome of methyl-cholanthrene-transformed mouse sarcoma cells (Shih et al., 1979). When taken together, these discoveries drove home the notion that cancer was a disease of oncogenes that, even when present in single copies per cell, could overwhelm the resident genomes of normal cells and dictate the subsequent phenotype of these cells, specifically the phenotype of neoplastic transformation.

There were, to be sure, a small number of dissident voices barely heard above the clamor of oncogene research. Henry Harris at Oxford University had developed a method of using Sendai virus to fuse cells in 1965 (Harris and Watkins, 1965). Four years later, he reported in *Nature* that fusion of certain cancer cells with certain non-neoplastic cells yielded hybrid cells of mixed genotype that were themselves non-tumorigenic (Harris et al., 1969). From this, he drew the conclusion that the phenotype of neoplastic transformation was a recessive trait, and that the cancer-causing genes present in the neoplastic cells lost their ability to drive cell transformation when confronted with the alleles present in the genomes of normal cells. Hence, cancer was a recessive trait, not the dominant trait. Harris ensured me at a meeting held in London in the early 1980s that the notion that cancer genes acted as dominant alleles, as well as the oncogene research—all the rage at the time—was wrong-headed and that many would regret jumping aboard the oncogene bandwagon.

The other voice in the field was that of Alfred Knudson. By examining the rates of retinoblastoma development in children, in 1971 he concluded that two `events’ were required in order to trigger the tumor (Knudson, 1979). At the time, the nature of these events, ostensibly mutations, was unclear, i.e. did they activate or inactivate the two homologous copies of a single gene or did they act on two distinct genes? By 1973, both Knudson and David Comings in California had proposed that the `events’ leading to cancer often led to the inactivation of genes, and that the two events that Knudson had calculated led to the loss of the two copies of a gene that were needed to impose normal proliferative control on cells and were thus, to use Harris’ parlance, `malignancy-suppressing genes’ (Comings, 1973; Knudson et al., 1973). Comings also proposed in his 1973 paper that tumor viruses arose by extracting cancer-causing genes from the normal cellular genome—3 years before the landmark paper from the Varmus and Bishop Laboratory (Stehelin et al., 1976)!

**Figure 1:**
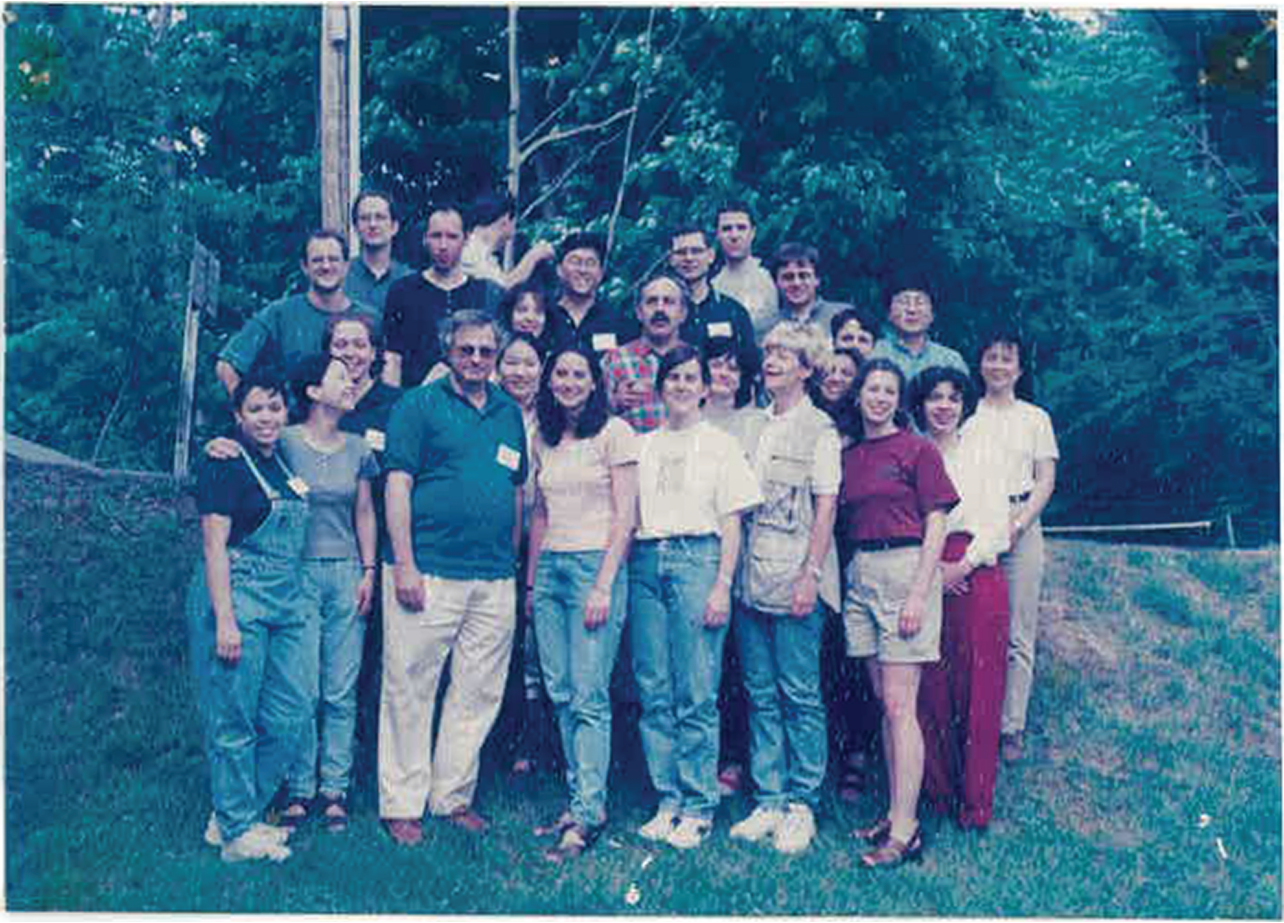
A group picture of the attendees at a joint Levine–Weinberg laboratory retreat in northwest Connecticut in the spring of 1998.

In my own group, the use of gene transfer, i.e. transfection, had led to the discovery of multiple transfectable oncogenes but in 1983 led to another insight: while a *RAS* oncogene introduced into cells via calcium phosphate-mediated transfection would readily transform NIH3T3 cells into tumor cells, it failed, in the hands of Hartmut Land, to do so when introduced into normal rat embryo fibroblasts (Land et al., 1983a). This led, in turn, to the speculation that immortalized cells, such as the NIH3T3 cells, had undergone a change that rendered them responsive and susceptible to introduced *RAS* oncogenes, while primary cells such as the REFs, which had not undergone immortalization, were not so equipped.

Land, working with Luis Parada, then demonstrated that the transformation of REFs might succeed if two oncogenes were introduced concomitantly, initially the *RAS* and the *MYC* oncogenes (Land et al., 1983a). The resulting phenomenon of `oncogene collaboration’, also revealed by H. Earl Ruley between *RAS* and the adenovirus *E1A* oncogene (Ruley, 1983), could then be generalized, suggesting that there was a requirement of one `cytoplasmic oncogene’, i.e. one whose gene product was localized to the cytoplasm with one `nuclear oncogene’, which made a nucleus-localized protein (Land et al., 1983b).

This set the stage for extending and generalizing this work. One attractive possibility was that *RAS* could collaborate with *TP53*, an idea inspired by the fact that both MYC and p53 were nuclear proteins, that they both seemed to be metabolically unstable and turned over at a rapid pace, and that they appeared to be expressed at higher levels in rapidly growing cells (including tumor cells) than within cells that had entered into a quiescent state. Its high-level expression in a variety of cancer cell types was, on its own, compelling testimony that p53 functioned as an oncoprotein, much like MYC!

The logic of this hypothesis was fulfilled beautifully in our 1984 paper as well as in an accompanying paper in *Nature*; both papers used a cloned *TP53* gene and found strong collaboration with *RAS* when introduced into REFs (Eliyahu et al., 1984; Parada et al., 1984). Moreover, the *TP53* gene had no effect on already-immortalized cells, leading to the notion that *RAS* was a `transforming’ oncogene and *MYC* and *TP53* were `immortalizing’ oncogenes. These two properties were thought to complement one another functionally, leading to the observation of oncogene collaboration.

However, there was a fly in the ointment. One disruptive finding was already reported by Varda Rotter and later her research group. In 1980, she reported that the `p50’ protein, now known to be p53, was present at very low concentrations in Abelson murine leukemia virus-transformed cells (Rotter et al., 1980). This was followed up in 1984 (Wolf et al., 1984), when her group reported that a line of Abelson leukemia virus-transformed cells had actually lost p53 function entirely through the gene-disrupting effects of an integrated retrovirus provirus. This work, on its own was essentially impossible to reconcile with the then-reigning hypothesis that p53 was a proto-oncogene.

In fact, there was another inconvenient truth in the logic underlying the p53-oncogene hypothesis, and it came from the sequencing of *TP53* clones—at the time a not-inconsiderable amount of work: early in 1988, the group of Arnie Levine, whose career we celebrate now, collaborating with that of Moshe Oren, described the fact that previously used clones of *TP53* actually contained subtle mutations, notably point mutations, while the clones of *TP53* that had been used in the oncogene collaboration experiments contained, with all likelihood, wild-type sequences (Finlay et al., 1988). Hence, the oncogenic powers of *TP53* actually derived from mutant alleles of *TP53.* This discovery and the subsequent findings reported the next year from the Levine and Oren groups that wild-type *TP53* could actually actively suppress transformation (Eliyahu et al., 1989; Finlay et al., 1989) sealed its fate: *TP53* was, following detailed functional characterization, a *bona fide* tumor suppressor gene!

This rather tortuous experimental trajectory may or may not teach object lessons about how to conduct experimental science. Perhaps initial preconceptions that were fulfilled by reasonably well-conducted experiments were too satisfying to examine critically, at least for several years. Perhaps, the only object lesson that clearly survives from this account derives from ancient history: `For want of a nail a kingdom fell’, i.e. the affairs of the world rise or fall depending on small details. Perhaps it is overly grandiose to analogize point mutations in *TP53* with horseshoe nails. Still, the argument survives: minute details, such as single-base changes, can radically change our perceptions of far larger problems, notably the distinction in this case between *bona fide* oncogenes and tumor suppressor genes.

## References

[ref1] Comings D.E. (1973). A general theory of carcinogenesis. Proc. Natl Acad. Sci. USA 70, 3324–3328. 4202843 10.1073/pnas.70.12.3324 PMC427229

[ref2] DeLeo A.B. (1998). p53-based immunotherapy of cancer. Crit. Rev. Immunol. 18, 29–35. 9419445 10.1615/critrevimmunol.v18.i1-2.40

[ref3] DeLeo A.B., Jay G., Appella E., et al. (1979). Detection of a transformation-related antigen in chemically induced sarcomas and other transformed cells of the mouse. Proc. Natl Acad. Sci. USA 76, 2420–2424. 221923 10.1073/pnas.76.5.2420 PMC383613

[ref4] Dippold W.G., Jay G., DeLeo A.B., et al. (1981). Transformation-related protein: detection by monoclonal antibody in mouse and human cells. Proc. Natl Acad. Sci. USA 78, 1695–1699. 6940183 10.1073/pnas.78.3.1695 PMC319199

[ref5] Eliyahu D., Michalovitz D., Eliyahu S., et al. (1989). Wild-type p53 can inhibit oncogene-mediated focus formation. Proc. Natl Acad. Sci. USA 86, 8763–8767. 2530586 10.1073/pnas.86.22.8763 PMC298370

[ref6] Eliyahu D., Raz A., Gruss P., et al. (1984). Participation of p53 cellular tumor antigen in transformation of normal embryonic cells. Nature 312, 646–649. 6095116 10.1038/312646a0

[ref7] Finlay C.A., Hinds P.W., and Levine A.J. (1989). The p53 proto-oncogene can act as a suppressor of transformation. Cell 57, 1083–1093. 2525423 10.1016/0092-8674(89)90045-7

[ref8] Finlay C.A., Hinds P.W., Tan T.-H., et al. (1988). Activating mutations for transformation by p53 produce a gene product that forms an hsc70-p53 complex with an altered half-life. Mol. Cell. Biol. 8, 531–539. 2832726 10.1128/mcb.8.2.531-539.1988 PMC363177

[ref9] Harris H., Miller O.J., Klein G., et al. (1969). Suppression of malignancy by cell fusion. Nature 223, 363–368. 5387828 10.1038/223363a0

[ref10] Harris H., and Watkins J.F. (1965). Hybrid cells derived from mouse and man: artificial heterokaryons of mammalian cells from different species. Nature 205, 640–646. 14287398 10.1038/205640a0

[ref11] Knudson A.G. Jr. (1979). Mutagenesis and embryonal carcinogenesis. Natl Cancer Inst. Monogr. 51, 19–24. 225668

[ref12] Knudson A.G. Jr, Strong L.C., and Anderson D.E. (1973). Heredity and cancer in man. Prog. Med. Genet. 9, 113–158. 4351406

[ref13] Land H., Parada L.F., and Weinberg R.A. (1983a). Tumorigenic conversion of primary embryo fibroblasts requires at least two cooperating oncogenes. Nature 304, 596–602. 6308472 10.1038/304596a0

[ref14] Land H., Parada L.F., and Weinberg R.A. (1983b). Cellular oncogenes and multistep carcinogenesis. Science 222, 771–778. 6356358 10.1126/science.6356358

[ref15] Lane D.P., and Crawford L.V. (1979). T antigen is bound to a host protein in SV40-transformed cells. Nature 278, 261–263. 218111 10.1038/278261a0

[ref16] Linzer D.I., and Levine A.J. (1979). Characterization of a 54K Dalton cellular SV40 tumor antigen present in SV40-transformed cells and uninfected embryonal carcinoma cells. Cell 17, 43–52. 222475 10.1016/0092-8674(79)90293-9

[ref17] Parada L.F., Land H., Weinberg R.A., et al. (1984). Cooperation between gene encoding p53 tumour antigen and *ras* in cellular transformation. Nature 113, 649–651. 10.1038/312649a0 6390217

[ref18] Rotter V., Witte O.N., Coffman R., et al. (1980). Abelson murine leukemia virusinduced tumors elicit antibodies against a host protein, p50. J. Virol. 36, 647–555. 10.1128/jvi.36.2.547-555.1980 PMC353673 6159484

[ref19] Ruley H.E. (1983). Adenovirus early region 1A enables viral and cellular transforming genes to transform primary cells in culture. Nature 304, 602–606. 6308473 10.1038/304602a0

[ref20] Shih C., Shilo B.Z., Goldfarb M.P., et al. (1979). Passage of phenotypes of chemically transformed cells via transfection of DNA and chromatin. Proc. Natl Acad. Sci. USA 76, 5714–5718. 230490 10.1073/pnas.76.11.5714 PMC411720

[ref21] Stehelin D., Varmus H.E., Bishop J.M., et al. (1976). DNA related to the transforming gene(s) of avian sarcoma virus is present in normal avian DNA. Nature 260, 17–173. 176594 10.1038/260170a0

[ref22] Wolf D., Harris N., and Rotter V. (1984). Reconstitution of p53 expression in a nonproducer Ab-MuLV-transformed cell line by transfection of a functional p53 gene. Cell 38, 119–126. 6088057 10.1016/0092-8674(84)90532-4

